# Experimental Study on the Skidding Damage of a Cylindrical Roller Bearing

**DOI:** 10.3390/ma13184075

**Published:** 2020-09-14

**Authors:** Qing Zhang, Jun Luo, Xiang-yu Xie, Jin Xu, Zhen-huan Ye

**Affiliations:** 1 School of Mechanical Engineering, Guizhou University, Guiyang 550025, China; zhangqingcumt@cumt.edu.cn; 2 Guizhou Provincial College-Based Engineering Research Center for Materials Protection of Wear and Corrosion, College of Chemistry and Materials Engineering, Guiyang University, Guiyang 550005, China; hx0040@gyu.edu.cn; 3 School of Engineering, Zunyi Normal College, Zunyi 563000, China; yzh@zync.edu.cn

**Keywords:** cylindrical roller bearing, skidding damage, microscopic analysis, real-time detection

## Abstract

As large-scale rotating machines develop toward high rotating speed and high power–weight ratio, skidding damage has become one of the major initial failure modes of cylindrical roller bearings. Therefore, understanding the skidding damage law is an effective way to ensure the safety of machines supported by cylindrical roller bearings. To realize the skidding damage, a high-speed rolling bearing test rig that can simulate the actual operating conditions of aviation bearings was used in this paper, and the skidding damage dynamic behaviors of cylindrical roller bearings were investigated. In addition, to ensure the accuracy of the obtained skidding damage mechanism, the cylindrical roller bearing was carefully inspected by microscopic analysis when the skidding damage occurred. Out results show that instantaneous increases in friction torque, vibration acceleration, and temperature are clearly observed when the skidding damage occurs in the cylindrical roller bearing. Furthermore, under the conditions of inadequate lubrication and light load, the critical speed of skidding damage is rather low. The major wear mechanisms of skidding damage include oxidation wear, abrasive wear, and delamination wear. The white layers are found locally in the inner ring and rollers under the actions of friction heat and shear force.

## 1. Introduction

As an important support of large-scale rotating machines, the performance and reliability of cylindrical roller bearings directly affect the safe performance of the machines. The *DN* value of a rolling bearing (inner diameter (mm) × speed (r/min)) increases continuously along with the large-scale rotating machines develop toward high speed and high power–weight ratio. The traction forces acting on the rollers and cage are not large enough to overcome the towing forces in the condition of a high DN value, and in this situation, slip occurs. Slip will aggravate the friction between the rollers and the inner ring. Furthermore, the friction heat generated by slip will deteriorate the lubrication of the bearing and eventually lead to the failure of the bearing caused by skidding damage [1,2]. According to statistics [3], about 34.46% of the failure rear bearings (cylindrical roller bearings) on the aero-engine spindles are caused by the skidding damage, indicating that skidding damage has become one of the most important failure modes of cylindrical roller bearings. Therefore, it is extremely urgent to investigate the skidding damage of rolling bearings.

Due to the complexity of the contact, motion relationship, and lubrication of rolling bearings, previous research mainly focused on the theoretical analysis of slip. Based on the Jones’ quasi-static method [4,5], a quasi-static analysis model was established by Harris [6], and the factors which can pose influence on the cage slip rate were analyzed. In order to quantify steady-state bearing operation, the SHABERTH computer program supplemented with skid data and experimental temperature was used by Poplawski [7]. The method is conducive to designing the system of hybrid ball thrust bearings. Yoshida [8] established an analytical method which enables the estimation of the cage slip and roller slip for a given bearing geometry and a given operating condition. A six degree of freedom dynamic bearing model was established by Kang [9], and the slip characteristics of the bearing under the conditions of the low load, transient acceleration, and deceleration were analyzed. Liao et al. [10] obtained the contact force and contact angle of a ball bearing through a geometric and force balance analysis. Moreover, they investigated the slip behavior of the ball bearing considering the simultaneous effects of radial and axial loads according to Hirano’s rule [11]. Laniado et al. [12] studied the relative slip between a rolling element and a raceway of the cylindrical roller bearing via a finite element simulation analysis. Jain et al. [13] established the dynamic model for angular contact ball bearings, and considering the influences of the centrifugal force and the gyro force, they further investigated the slip characteristics of the bearings under the simultaneous effects of radial and axial loads.

To the best of our knowledge, few research works have studied the slip experimentally until now. Selvaraj et al. [14] investigated the effects of operating parameters, including radial load, viscosity of lubricating oil, speed, the number of rollers, and the temperature of bearing on the cage slip by a self-made test machine. They found that an increase of the speed and a decrease of the radial load will increase the slip rate of bearing, and appropriately increasing the temperature will reduce the slip rate. Subsequently, Li et al. [15] built a bearing tester in the process of studying the influence of the test parameters on the friction power consumption of high-speed rolling bearings, and they found that the overall friction power consumption of the bearing shows an increasing trend with an increase in the slip rate. The minimum load against slip and the maximum critical load against the overheating of the rolling bearing were analyzed by Cui et al. [16] based on a self-developed high-speed rolling bearing tester.

Because the ultimate failure mode of the rolling bearing is skidding damage, it is difficult to solve this problem only by studying the slip. Since the skidding damage in rolling bearing usually demonstrates smearing and burning damage [17], some researchers investigated the smearing damage of rolling bearings. Cocks et al. [18] studied the effects of the rolling speed, sliding speed, oil flow rate, ambient temperature, rate of increase in load, and ball surface roughness on the smearing damage of a rolling bearing with a two-ball sliding tester. They found that an elastohydrodynamic film of lubricant plays a major role in determining the load when the smearing damage occurred. The changes of the mechanical characteristics, friction coefficient, frictional power intensity, elastohydrodynamic film thickness, and temperature between the roller and raceway of a cylindrical roller bearing after the smearing damage were investigated by Mark et al. [19] based on a twin-roll test rig. Additionally, the anti-smearing damage abilities of four kinds of surface coating/modified layers were evaluated by Evans et al. [20] using a four-axis testing machine, and they concluded that WC/a-C:H coating shows an obvious effect on anti-bearing smearing damage. Li et al. [17] studied the mechanism of smearing damage of the rolling bearings caused by different slip combinations using a roll-slip test rig. It was concluded that reducing the slip rate and the speed of inner ring are beneficial for reducing the slip degree of rolling bearings. However, the contact processes between a roller and an inner ring simulated by these test devices are inconsistent with the actual running conditions of rolling bearings.

From the foregoing, a large number of people have studied the slip with theory due to the lack of test conditions and the complex internal contact relationship of rolling bearings; unfortunately, it is not the slip but the skidding damage that is the final failure mode of the bearing. Few have studied skidding damage by the test rigs, however, the test conditions adopted by them are inconsistent with the actual working conditions of rolling bearings. In this paper, the skidding damage of cylindrical roller bearings was investigated by a high-speed rolling bearing test rig, which could simulate the actual working conditions of the bearing. In addition, the slipping and skidding damage dynamic behavior of cylindrical roller bearings were investigated by the test rig. Furthermore, the major skidding damage wear mechanisms for cylindrical roller bearings were acquired by microscopic analysis. Therefore, all the work done in this article is meaningful for skidding damage protection and prolonging the service life of rolling bearings in the domains of aeroengine, wind turbine, and other large rotating machines.

## 2. Experimental Details

### 2.1. Test Bearing

**Figure 1:**
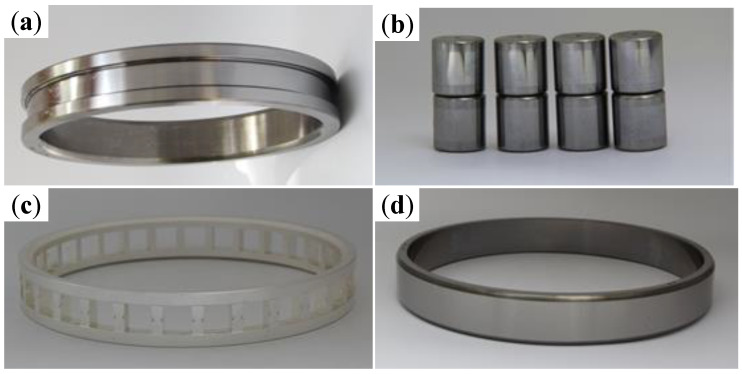
The test bearing: (**a**) inner ring; (**b**) rollers; (**c**) cage; (**d**) outer ring.

The bearing used in the test consists of an inner ring, a certain number of rollers, a cage, and an outer ring. The inner ring of the bearing contains double stop edges, the outer ring has no stop edges, and each bearing contains 30 rollers, as shown in Figure 1. The detailed structural parameters of the test bearing are shown in Table 1. The inner ring, outer ring, and rollers are made of Cr4Mo4V bearing steel, and the content of each chemical element is shown in Table 2. The cage is made of silicon bronze with silver plating on the surface. In addition to silicon, the cage also contains a small amount of other alloying elements such as zinc, iron, and so on. The detailed compositions of the cage are shown in Table 3.

### 2.2. Experimental Equipment and Methods

**Figure 2:**
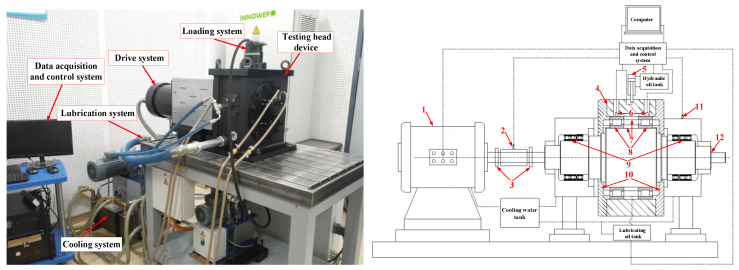
The high-speed rolling bearing test rig: (1) Induction motor; (2) Torque sensor; (3) Coupling; (4) Sealed cavity; (5) Hydraulic loading device; (6) Temperature sensor; (7) Retaining ring; (8) Test bearing; (9) Support bearing; (10) Positioning flange; (11) Vibration sensor; (12) Spindle.

All experiments were performed on the high-speed rolling bearing test rig in the State Key Laboratory of Tribology at Tsinghua University, Beijing, China. As shown in Figure 2, the test rig is mainly composed of a drive system, a cooling system, a loading system, a testing head device, a lubrication system, and a data acquisition and control system. The inner rings of the two test bearings, separated by a retaining ring, are hot mounted at the center of the main shaft with an interference fit. The outer rings, separated by a retaining ring, are cold mounted at the inner side of the sealing cavity with an interference fit. The positioning flanges on both sides of the main shaft and the sealing cavity are used to locate the inner and outer rings of the two bearings axially. Lubricating oil enters the sealing cavity to cool and lubricate the test bearings. Then, the oil flows into the testing head cavity through the oil drain hole under the positioning flanges and it returns the oil tank through the oil pipe. The motor drives the spindle, which is supported by the support bearings on both sides (three on each side) in the testing head cavity, to rotate through the couplings. The motor, lubricating oil tank, and supporting bearings on both sides are cooled by the water. During the tests, the spindle drives the inner rings of the two test bearings to rotate, and the outer rings are fixed (the sealing cavity is fixed on the cavity of the testing head). The rotational speed, radial load, and oil intake are controlled and monitored in real time by the data acquisition and control system. Moreover, the friction torque, vibration acceleration, temperature of the test bearings, and lubricating oil temperature are also monitored in real time by the same system. The range of speed (*N*) is 0–2.0 × 10^4^ rpm, the range of radial load (*F*) is 0–5.0 KN, the range of speed acceleration (a_n_) is 0–4.0 × 10^6^ rpm/h, the range of oil intake (*L*) is 0–4.0 L/min, and the load acceleration (a_f_) is 6.0 × 10^3^ KN/h.

Table 4 displays the concrete parameters of each experiment. After testing, the microstructure of the skidding damage surface of the test bearing was observed by a SEM (Scanning Electron Microscope (FEI Quanta 250 FEG, Hillsboro, OR, USA)). The section metallographic organization was observed by an OM (Optical Microscope (ZEISS Axio Vert.Al, Jena, Germany)). In addition, the chemical component was obtained by EDX (Energy Dispersive X-Ray Spectroscopy (FEI Quanta 250 FEG, Hillsboro, OR, USA) and Raman spectrometer (SENTERRA, Karlsruhe, Germany). The profile was measured by the non-contact 3D surface optical profilometer (Trimos TR-Scan, Suzhou, China). The hardness of the metallographic structure was measured by a Vickers hardness tester.

## 3. Results and Discussions

### 3.1. Realization of the Skidding Damage

**Figure 3:**
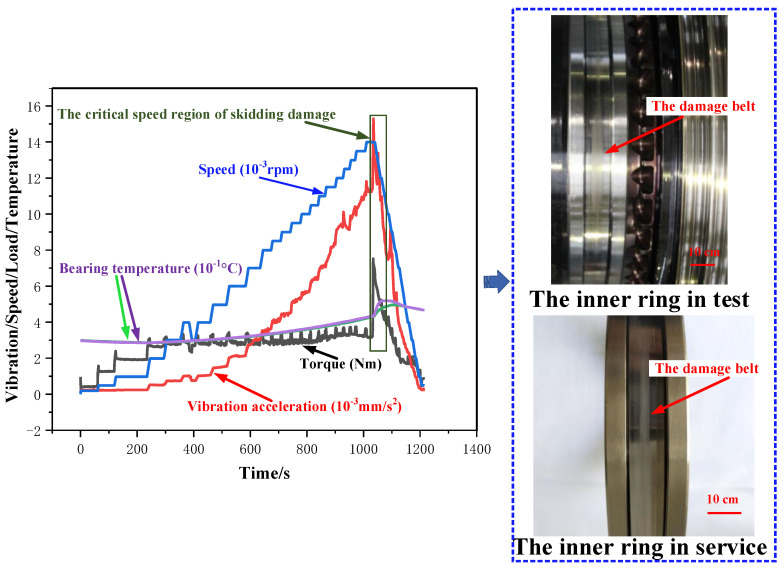
Dynamic behavior curve of the skidding damage. (*F* = 0.2 KN, *L* = 2.166 L/min, a_n_ = 3.6 × 10^5^ rpm/h).

The dynamic behavior curve of the skidding damage of the cylindrical roller bearing is shown in Figure 3. The radial load, oil intake, and acceleration were set as constants, and the speed was increased continuously at the same acceleration value during the test. The friction torque, vibration acceleration, and temperature of the bearings had an instantaneous increase synchronously, and the entire testing head made a large abnormal noise when the speed reached approximately 13,980 rpm. Then, the speed was decreased to zero with the same acceleration and the testing head was disassembled to investigate the test bearings. The black-gray banded wear areas (also known as the damage belt), the two ends of which are shaped like broom, are found in the inner ring of a test bearing, which is consistent with the macroscopic appearance of the inner ring on a bearing that failed by skidding damage that occurs during the service life of certain types of aeroengines. Therefore, it was concluded that the cylindrical roller bearing suffered from skidding damage during the test. According to the bearing dynamic behavior curve, the critical speed for skidding damage can be identified by the sudden and synchronous increases of the friction torque, vibration acceleration, and temperature.

The centrifugal force on the roller makes it move away from the inner ring to the outer when the bearing runs at high speed, which causes the decrease of the dragging force of the oil film between the roller and inner ring and lead to the slip ultimately. The slip will aggravate the friction between the roller and inner ring, and thus the internal friction heat of the bearing increases dramatically. Accordingly, the viscosity of the lubricating oil decreases, the material softens, and eventually the lubricating oil film is fractured.

**Figure 4:**
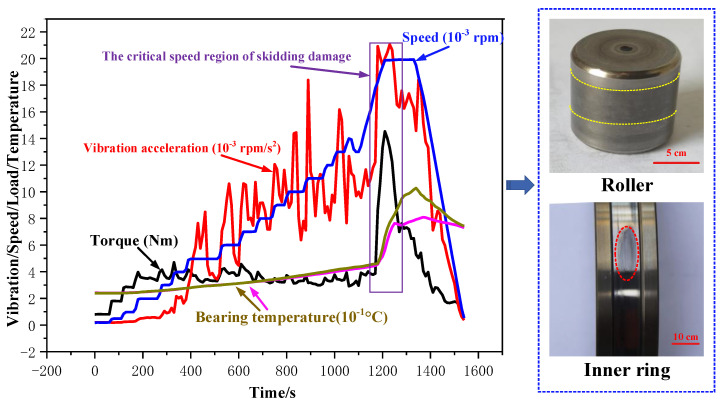
Dynamic behavior curve of the skidding burn (*F* = 1 KN, *L* = 4.332 L/min, a_n_ = 3.6 × 10^5^ rpm/h).

The dynamic behavior curve of the skidding burn is shown in Figure 4. The radial load, oil intake, and acceleration were set as constants, and the speed was increased continuously at the same acceleration during the test. After the cylindrical roller bearing suffered from the skidding damage, the speed was further increased to 2.0 × 10^4^ rpm and then decelerated to zero after running for a period of time. The maximum temperature of a cylindrical roller bearing rose to 102.8 °C. After checking the bearing, it was found that the inner raceway appears irregular oval-shaped black burning areas, which intermittently surround the whole circumference of the inner ring, and the rollers are basically dark black. Therefore, the cylindrical roller bearing has suffered from the skidding burn during the test.

### 3.2. Slipping Dynamic Behavior of the Cylindrical Roller Bearing

**Figure 5:**
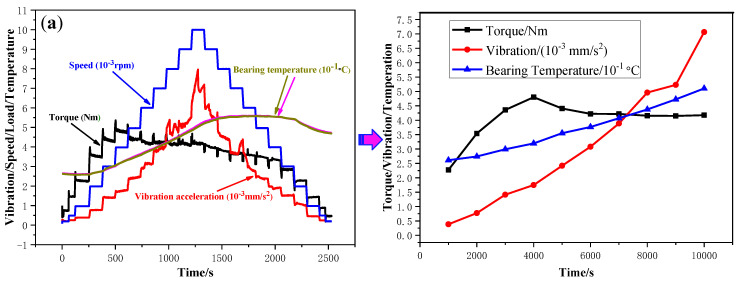

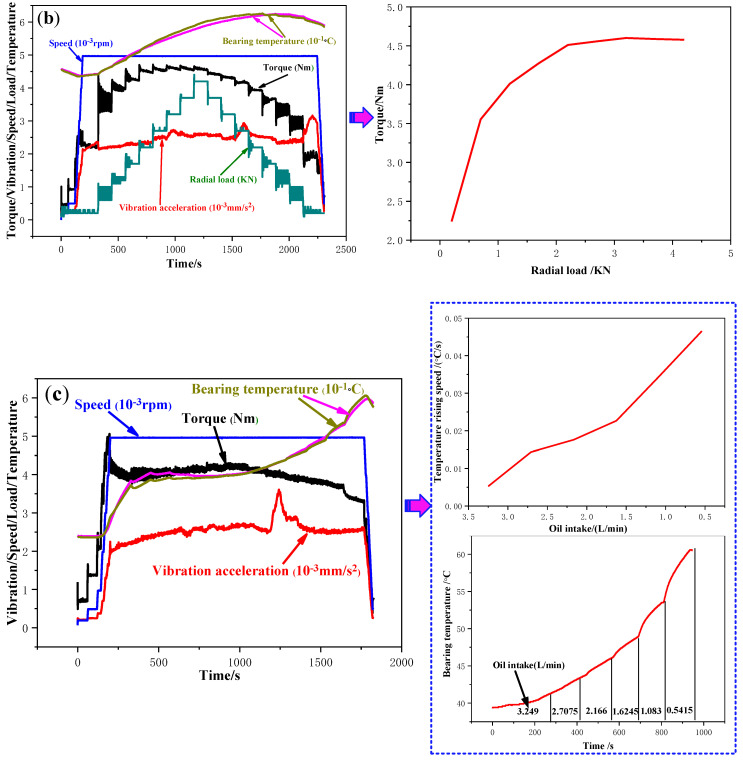

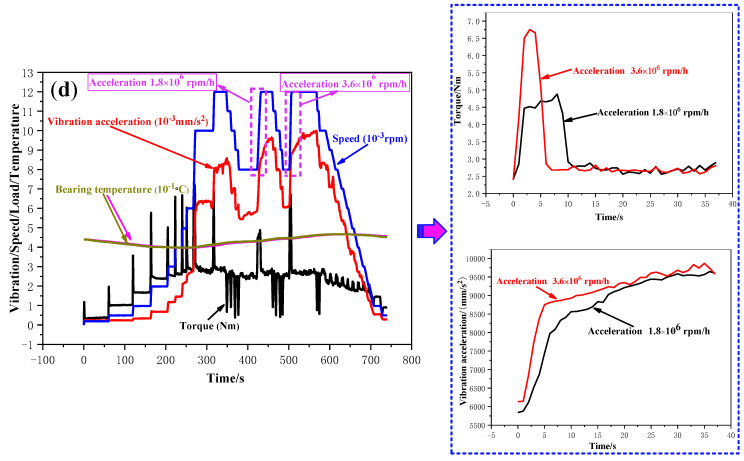
Dynamic behavior of the cylindrical roller bearing for different operating conditions. (**a**) Speed; (**b**) Radial load; (**c**) Oil intake; (**d**) Speed acceleration.

In order to investigate the influence of the speed on the slipping dynamic behavior of the cylindrical roller bearing, the radial load and oil intake were controlled at the unchanged values and only the speed was changed at the same acceleration value. The changes of the friction torque, vibration acceleration, and temperature were recorded. The dynamic behavior curve is shown in Figure 5a.

The temperature of the cylindrical roller bearing shows linear-like increasing trend along with the increase of the speed, as shown in Figure 5a. This probably due the fact that a decrease of the dragging force of the inner ring on the roller and an increase of the friction resistance from the lubricating oil with the continuous increase of the speed, which together intensify the relative slip between the rollers and the inner ring. In addition, the increase of the speed also increases the friction between the roller and the raceway, which results in an increase of the bearing temperature. The friction torque of the cylindrical roller bearing increases along with the increase of the speed; moreover, the value of friction torque starts to decrease and finally tends to be stable when the speed exceeds a certain value. This can be ascribed to the fact that the lubrication between the inner ring and the rollers is insufficient at low speed, and the two parts contact each other directly. Therefore, the friction torque increases with the increase of the speed at first. After the inner ring and rollers are fully lubricated, the friction torque decreases eventually and tends to stable at last. The vibration acceleration increases steadily along with the increase of the speed, and it should be noted that the vibration acceleration increases sharply when the speed higher than a specific value. This may be because the collision speed between the rollers and the raceway increases with the increase of the speed. Furthermore, the vibration acceleration will extremely increase at high speed due to the nonuniformity of the bearing quality.

In order to investigate the influence of the radial load on the slipping dynamic behavior of the cylindrical roller bearing, the speed and oil intake were controlled at unchanged values and only the radial load was changed at the same acceleration, as it is shown in Figure 5b.

It can be seen that the radial load mainly affects the friction torque of the cylindrical roller bearing. The friction torque increases rapidly in the initial stage, and the increase trend slows down when the radial load exceeds a certain value. Probably, two items can be used to elaborate the phenomenon. One, the greater the radial load is, the larger the friction torque caused by elastic hysteresis loss is. Two, the friction torque caused by the contact load between the rollers and the cage also increases along with the increase of radial load. However, a nonlinear relationship can be identified between the radial load and friction torque, which is probably due to the fact that the amplitude of the contact deformation between roller and raceway become smaller and smaller with the increase of the radial load.

In order to investigate the influence of the oil intake on the slipping dynamic behavior of the cylindrical roller bearing, the speed and radial load were controlled at fixed values and only the oil intake was changed, and the results are shown in Figure 5c.

The oil intake mainly affects the temperature of the cylindrical roller bearing. The temperature presents an increasing trend and its rising speed accelerates with the decrease of the oil intake. This may be because the lower the oil intake is, the less sufficient the lubrication is. In addition, the generated heat will not be easily dissipated when the oil intake is inadequate.

A dynamic behavior curve of the speed acceleration and deceleration can be seen in Figure 5d. The radial load and the oil intake were controlled at unchanged values and only the speed acceleration was changed.

It can be seen that the friction torque and the vibration acceleration increase instantaneously along with the instantaneous increase of the speed. Then, the friction torque tends to be stable after rapidly decreasing, and the vibration acceleration also tends to be stable after slightly decreasing. This may be because the large speed difference between the rollers and the inner ring causes the frictional resistance on the rollers to increase instantaneously in this process, then, the resistance decreases to a stable value when the speed is stable. In addition, the friction torque, vibration acceleration, and bearing temperature decrease instantaneously, and the torque even turns into a negative value in the process of the instantaneous decline of the speed. This may be because the huge reverse speed difference between the inner ring and the rollers makes the friction torque decrease instantly and even to the opposite direction during the instantaneous decrease of the speed. The changes of the friction torque and the vibration acceleration are compared when the speeds increase under the two accelerations (1.8 × 10^6^ rpm/h and 3.6 × 10^6^ rpm/h). It is found that the greater the acceleration is, the faster the instantaneous increases of the friction torque and the vibration acceleration are, and the greater the maximum friction torque is. This may be because the greater the acceleration is, the greater the speed difference between the inner ring and the rollers is at the same starting and ending speed, which makes it easier for the rollers to collide with the cage, as well as the inner and outer rings. The two parameter values have little difference under different accelerations after the speed is stable.

### 3.3. Skidding Damage Dynamic Behavior of the Cylindrical Roller Bearing

**Figure 6:**
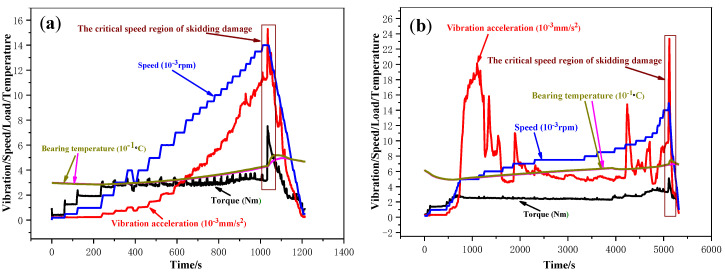

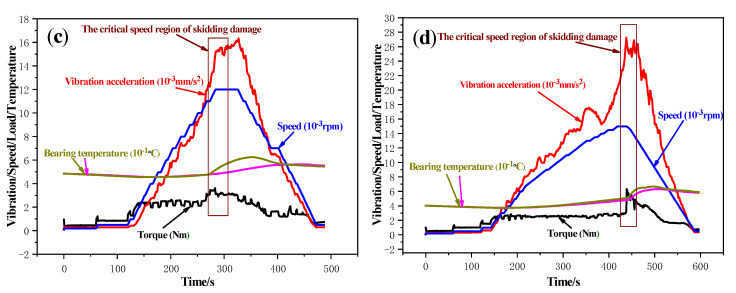
Skidding damage dynamic behavior curves of the cylindrical roller bearings under the four working conditions. (**a**) *F* = 0.2 KN, *L* = 2.166 L/min; (**b**) *F* = 0.2 KN, *L* = 0.5415 L/min; (**c**) *F* = 1 KN, *L* = 0.5415 L/min; (**d**) *F* = 1 KN, *L*= 2.166 L/min.

To investigate the skidding damage of the cylindrical roller bearing, four series of tests were conducted, and all the test speeds were increased to critical speeds (Table 5). Moreover, the radial loads and oil intakes were also changed in the tests, and the detailed parameters are shown in Table 5. The dynamic behavior curves are shown in Figure 6.

In the tests NO. 1 and NO. 2, the critical speed lowered from 13,980 rpm to 11,050 rpm due to the reduction in the oil intake from 2.166 L/min to 0.5415 L/min. This may be because that the lubricating oil films between the rollers and the inner ring are easier to be fractured during the high-speed operation due to a decrease of the size of film and the worsening of the cooling effect with the decrease in the oil intake.

Compared the test NO.2 with NO.3, the critical speed reduced from 14,961 rpm to 11,050 rpm because the radial load was reduced from 1 KN to 0.2 KN. Under lower radial load, it is more likely to slip due to the dragging force of oil film on the roller and overcoming the sum of the resistances on it becomes difficult. The negative impact of the slip gets more and more serious with the continuous increase of the speed, and thus, the skidding damage occurs at relatively low speed. Therefore, the critical speed of skidding damage is lower under a light load than that under a heavy load.

The critical speed of skidding damage was reduced from 14,961 rpm to 11,050 rpm, a reduction of 3911 rpm or 26.14%, due to the fact that the radial load was reduced from 1 KN to 0.2 KN when the oil intake was 0.5415 L/min in comparison to the second and third test conditions. However, the critical speed was reduced from 14,940 rpm to 13,980 rpm, a reduction of 960 rpm or 6.43%, because the radial load was reduced from 1 KN to 0.2 KN when the oil intake was 2.166 L/min in comparison to the first and fourth test conditions. The result indicates that increasing the oil intake can offset the influence of the light radial load on the critical speed of skidding damage for the cylindrical roller bearing.

### 3.4. Calculation of the Slip Rate of Cage Based on Pseudo-Dynamic Model

α—Contact angle of cylindrical roller bearing
χ—Displacement of the roller on the x-axis
*Φ*—Azimuth of roller
*ψ*—Orbital position angle of roller
*r_i_*—Angular velocity of roller at principal plane i
*D_g_*—Diameter of roller
*D_m_*—Diameter of bearing pitch circle
*D_w_*—Diameter of outer ring
*m*_2_—Short axis coefficient of contact ellipse
*ω_n_*—The rotational speed of inner ring
*ω_w_*—The rotational speed of outer ring
*ω_L_*—The theoretical rotational speed of cage
*ω_s_*—The actual rotational speed of cage
*ω_x_*—Angular velocity of roller around x-axis
*ω_y_*—Angular velocity of roller around y-axis
*ω_z_*—Angular velocity of roller around z-axis
*ω_o_*—Angular velocity of revolution of roller
*η_o_*—Viscosity of lubricating oil
*L*—Length of roller
*F*_1*i*_—Normal force of the i-th roller on the cage
*F*_2*i*_—Normal resistance of oil-gas mixture to the i-th roller
*f*_1*i*_—Tangential force of the i-th roller on the cage
*F_y_*—*y*-axis direction load
*F_z_*—*z*-axis direction load
*M_y_*—Moment around the *y*-axis
*M_z_*—Moment around the *z*-axis
*E_gw_*—Effective modulus of elasticity between roller and outer ring/(N/m^2^)
*E_gn_*—Effective modulus of elasticity between roller and inner ring/(N/m^2^)
*R_gw_*—Effective radius on the contact surface between roller and outer ring/m
*R_gn_*—Effective radius on the contact surface between roller and inner ring/m
*V_gwj_*—Average velocity of each point in the contact area between the j-th slice of the roller and the outer ring/(r/min)
*V_gnj_*—Average velocity of each point in the contact area between the j-th slice of the roller and the inner ring/(r/min)
*Q_wij_*—Contact force between roller and outer ring
*Q_nij_*—Contact force between roller and inner ring
*T_gnij_*—Dragging force between the j-th slice on the i-th roller and the inner ring
*T_gwij_*—Dragging force between the j-th slice on the i-th roller and the outer ring

Because of the complexity of the operating environment, it was difficult to measure the slip rate of the cage in real time during the test. Therefore, an effective pseudo-dynamic model [21,22] was adopted to measure the slip rate of the cage under different working conditions in this work.

**Figure 7:**
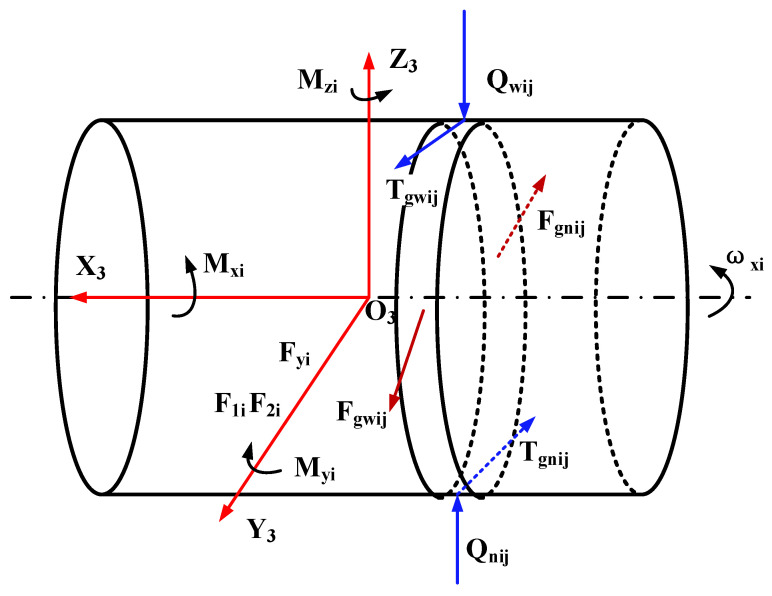
Schematic diagram of the forces and torques on the roller.

Figure 7 is a schematic diagram of the forces and torques on the roller of the cylindrical roller bearing. The pressures imposed by the inner and outer rings acting on the j-th slice of the i-th roller by means of the lubricating oil film are *F_gwij_* and *F_gnij_*, respectively [21], and the calculation formulas are as follows:

(1) $$\{\begin{array}{l} F_{gwij}=1.43LE_{gw}R_{gw}(1+\frac{D_{w}}{D_{m}}){\left(\frac{\eta_{o}V_{gwj}}{2E_{gw}R_{gw}}\right)}^{0.71}\\ F_{gnij}=1.43LE_{gn}R_{gn}(1-\frac{D_{w}}{D_{m}}){\left(\frac{\eta_{o}V_{gnj}}{2E_{gn}R_{gn}}\right)}^{0.71} \end{array}$$

The inertial moment acting on the i-th roller during the running of the bearing can be calculated by formula as follows:

(2) $$\{\begin{array}{l} M_{xi}=-\frac{m_{2}D_{w}^{2}}{8}{\dot{\omega }}_{xi}\\ M_{yi}=-\frac{m_{2}D_{w}^{2}}{8}{\dot{\omega }}_{yi}\\ M_{zi}=-\frac{m_{2}D_{w}^{2}}{8}{\dot{\omega }}_{zi} \end{array}$$

For the cylindrical roller bearing running smoothly, the inertial force on it can be obtained by following formula:

(3) $$\{\begin{array}{l} F_{yi}=m_{2}{\dot{\omega }}_{oi}r_{i}\\ F_{zi}=m_{2}\omega_{oi}^{2}r_{i} \end{array}$$

For the i-th roller of the cylindrical roller bearing, the following equilibrium equations can be established:

(4) $$\{\begin{array}{c} \frac{1}{n}\sum {}_{j=1}^{n}(T_{gwij}-T_{gnij}+F_{gwij}-F_{gnij})+F_{1i}+F_{2i}+F_{yi}=0\\ \frac{1}{n}\sum {}_{j=1}^{n}(Q_{nij}-Q_{wij})-f_{1i}+F_{zi}=0\\ \frac{D_{w}}{2}(\frac{1}{n}\sum {}_{j=1}^{n}(T_{gnij}+T_{gwij})-f_{1i})+M_{xi}=0\\ \frac{1}{n}\sum {}_{j=1}^{n}(Q_{nij}x_{j}-Q_{wij}x_{j})+M_{yi}=0\\ \frac{1}{n}\sum {}_{j=1}^{n}(F_{gnij}x_{j}-F_{gwij}x_{j})+M_{zi}=0 \end{array}$$

It is assumed that the rotating speed of the cage remains unchanged under certain working conditions, the equilibrium equation of the forces on the cage are as follows:

(5) $$\{\begin{array}{c} F_{g}\sin\psi -F_{t}\cos\psi +\sum {}_{i=1}^{N}(-F_{1i}\cos\phi_{i}+f_{1i}\sin\phi_{i})=0\\ \sum {}_{i=1}^{N}F_{1i}=0\\ F_{g}\cos\psi +F_{t}\sin\psi +\sum {}_{i=1}^{N}(F_{1i}\sin\phi_{i}+f_{1i}\cos\phi_{i})=0 \end{array}$$

The acting force of the rollers and cage on the inner ring of the bearing is the reaction force of the inner ring on the rollers and cage. The equilibrium equations of the inner ring are obtained by analyzing the forces on it:

(6) $$\{\begin{array}{c} F_{y}-F_{g}\sin\psi +F_{t}\cos\psi -\sum {}_{i=1}^{N}w\sum {}_{j=1}^{n}\frac{Q_{nij}}{L}\sin\phi_{i}=0\\ F_{z}-F_{g}\cos\psi -F_{t}\sin\psi -\sum {}_{i=1}^{N}w\sum {}_{j=1}^{n}\frac{Q_{nij}}{L}\cos\phi_{i}=0\\ M_{y}-\sum {}_{i=1}^{N}(w\sum {}_{j=1}^{n}\frac{Q_{nij}}{L})\frac{\sum {}_{j=1}^{n}Q_{nij}\left[(j-0.5)w-0.5L\right]}{\sum {}_{j=1}^{n}Q_{nij}}\cos\phi_{i}=0\\ M_{z}-\sum {}_{i=1}^{N}(w\sum {}_{j=1}^{n}\frac{Q_{nij}}{L})\frac{\sum {}_{j=1}^{n}Q_{nij}\left[(j-0.5)w-0.5L\right]}{\sum {}_{j=1}^{n}Q_{nij}}\sin\phi_{i}=0 \end{array}$$

Solution description: the fourth-order difference method was used to convert the differential equation system into a nonlinear equation system, and then the nonlinear equation system was divided into a plurality of small equations, which were solved by the steepest descent method and the Newton–Raphson method. The pseudo-dynamic model was used to calculate the actual rotational speed of the cage under various working conditions, and then the slip rate of cage was calculated to judge the slip degree of the bearing.

The computational formula of the theoretical rotational speed of cage is as follows:

(7) $$\omega_{L}=\frac{\omega_{n}(1-\gamma )+\omega_{w}(1+\gamma )}{2}$$

where: *r* = *D_g_/D_m_* cos α.

The formula calculating the slip rate of cage is as follows:

(8) $$S=\frac{\omega_{L}-\omega_{s}}{\omega_{L}}$$

The calculation results of the slip rate of cage based on the pseudo-dynamic model [21] are as showed in Table 6. With increases of speed and lubrication oil viscosity, and a decrease of radial load, the slip rate of cage shows an increasing trend. This is because the increase of rotational speed, the decrease of radial load (reducing the friction driving force on the roller), and the increase of lubrication viscosity (increasing the friction resistance on the roller) will increase the rotational speed difference between the inner ring and the turnover speed of the roller, therefore, the slip rate of the cage shows an increasing trend.

### 3.5. Microscopic Analysis of the Skidding Damage

**Figure 8:**
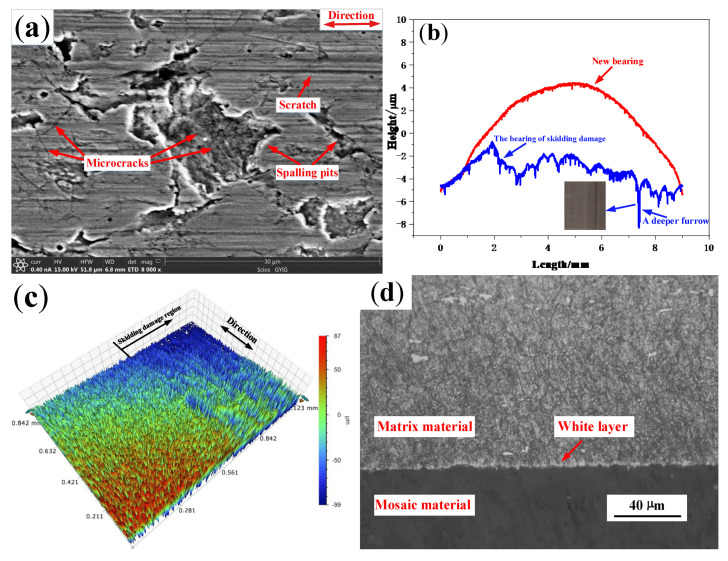
The damage surface of the inner ring on the skidding damage bearing (**a**) Microscopic topography; (**b**) Profile; (**c**) Three-dimensional topography; (**d**) Metallographic microstructure.

**Figure 9:**
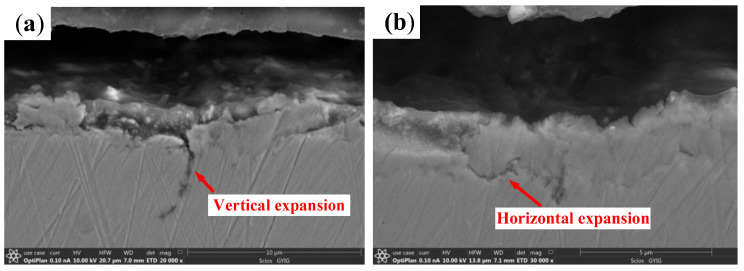
The cross-sectional topography of the damage belt on the inner ring. (**a**) Vertical crack; (**b**) Horizontal crack.

The skidding damage of cylindrical roller bearings are mainly reflected by surfaces of the inner ring raceways and rollers. As can be seen in Figure 8a, a large number of spalling pits with different sizes and microcracks are found on the surface of damage belt. Additionally, a deep furrow appears on the wear surface, and the maximum wear depth of the inner ring is about 8 µm. There are more scratches along the rolling direction in the damage belt as shown in Figure 8c. In Figure 8d, a white layer structure with a thickness of about 3–4 µm is found on the damage area of the inner ring. Furthermore, as shown in Figure 9, the crack of the vertical propagation has a depth of about 5 µm, and the horizontal propagation has a depth of about 2.5 µm and length of about 4 µm.

**Figure 10:**
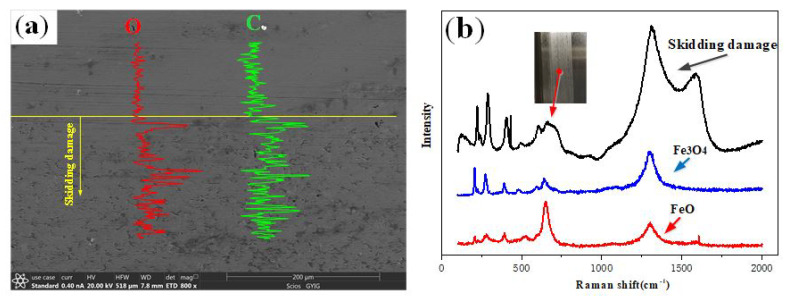
EDX line scanning-spectrum and Raman spectrum of the damage belt on the inner ring. (**a**) EDX line scanning-spectrum; (**b**) Raman spectrum.

The EDX scanning-pattern on the damage belt surface of the inner ring is shown in Figure 10a. The contents of C and O elements are obviously increased. The friction oxidation on the damage belt surface results in higher content of the O element. Furthermore, the Fe element is oxidized into FeO and Fe_3_O_4_ (Figure 10b) [23,24]. Therefore, the higher local flash temperature appears when the skidding damage happens because the FeO usually generated when the temperature is above 570 °C [23]. The increase of the C element is mainly due to the fact that a relatively larger amount of residual austenite appears on the damage belt surface, and the austenite contains larger amount of C elements than the non-austenite.

**Figure 11:**
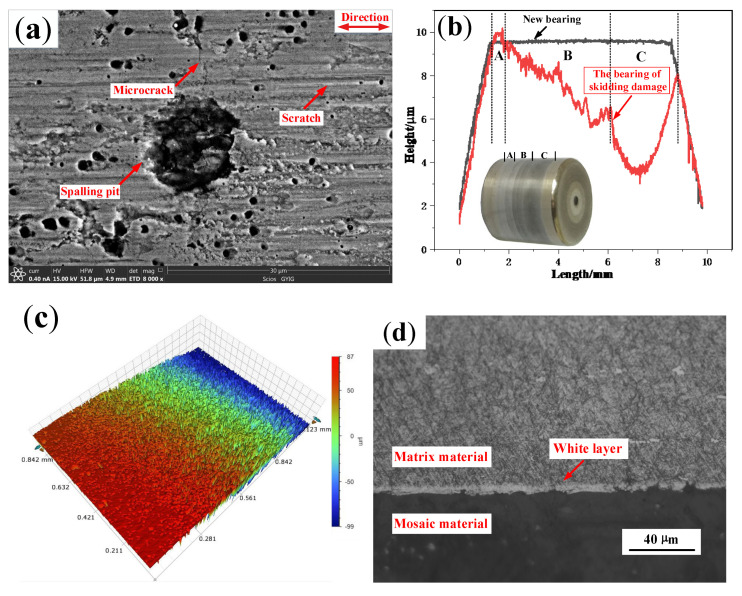
The damage surface of the roller on the skidding damage bearing. (**a**) Microscopic topography; (**b**) Profile; (**c**) Three-dimensional topography; (**d**) Metallographic microstructure.

**Figure 12:**
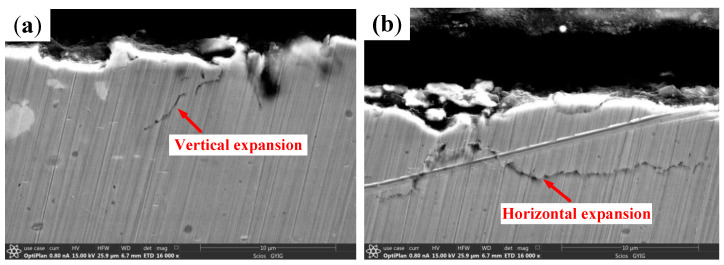
The cross-sectional topography of the damage area on the roller. (**a**) Vertical crack; (**b**) Horizontal crack.

The microstructure and profile of the wear area on the roller are shown in Figure 11. A large number of spalling pits, which are approximately circular with a diameter of around 13 µm, appear on the damaged surface. Moreover, microcracks and scratches along the rolling direction can also be found as shown in Figure 11a. The maximum wear depth of the roller is about 6.5 µm. Additionally, as shown in Figure 11b, the surface color is black, and the contour is seriously worn. As can be seen from Figure 11c, the roller surface is arc-shaped, and there are a large number of pits caused by spalling. The metallographic structure of the damaged area is shown in Figure 11d. A white layer structure with a thickness of about 7–8 µm appears on the damaged area of the roller. The white layer is easy to be fractured under an alternating load because of its brittleness. In addition, the cross-sectional topography of the damaged area is shown in Figure 12. It can be seen that the crack of the vertical propagation has a depth of about 7 µm, the included angle has a surface of about 45°, and the crack of the horizontal propagation has a depth of about 6 µm and a length of about 15 µm.

**Figure 13:**
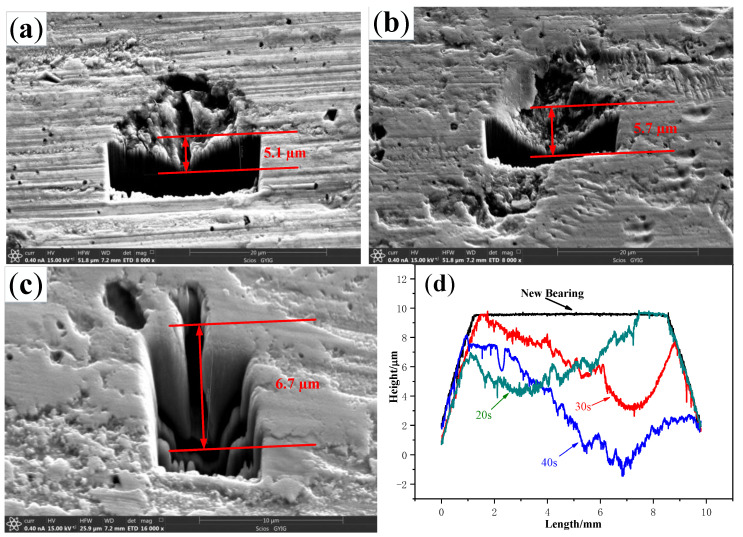
Comparison of the depth of spalling pits on the rollers. (**a**) 20 s; (**b**) 30 s; (**c**) 40 s; (**d**) Profile.

In order to investigate the effect of the operating time at critical speed on the damage of the bearing, the speed did not decrease immediately after the skidding damage occurred, but maintained the critical speed of skidding damage for 20 s, 30 s, and 40 s respectively. It was found that the maximum wear depths of the rollers are about 5.4 µm, 6.5 µm, and 11 µm respectively, and the depths of the typical spalling pits are about 5.1 µm, 5.7 µm, and 6.7 µm respectively, as shown in Figure 13.

The longer the running time of the cylindrical roller bearing at the critical speed of skidding damage is, the more serious the profile of roller wear. This may be because the surface finish of the roller is damaged and the coefficient of friction is increasing after the skidding damage happens, which accelerates the wear process. The longer the running time of the bearing at the critical speed is the deeper the typical peeling pit is. This may be because some materials in the contact area between the rollers and the inner ring are peeled off under the action of concentrated stress at the moment of skidding damage. The longer the bearing stays at the critical speed, the more severe the exfoliation based on the previous peeling pit is. Therefore, it is necessary to avoid the bearing running at or above the critical speed of skidding damage during the actual operation.

**Figure 14:**
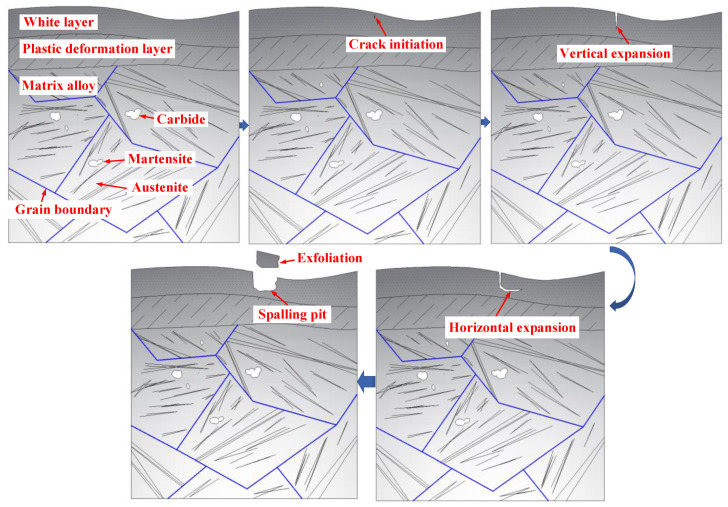
The spalling formation process of the damage area.

The spalling formation process of the cylindrical roller bearing can be inferred from the cross-sectional topography of the damage area on the bearing, as shown in Figure 14. The white layer is found locally on the bearing surface under the action of the friction heat and the shear force. Subsequently, vertical and horizontal cracks are formed in the white layer, and spalling occurs when the crack is close to the surface. It can be concluded that the deeper the vertical crack propagates, the deeper the spalling pit is. Therefore, the major wear mechanisms of the cylindrical roller bearing of skidding damage include oxidation wear, abrasive wear, and delamination wear.

**Figure 15:**
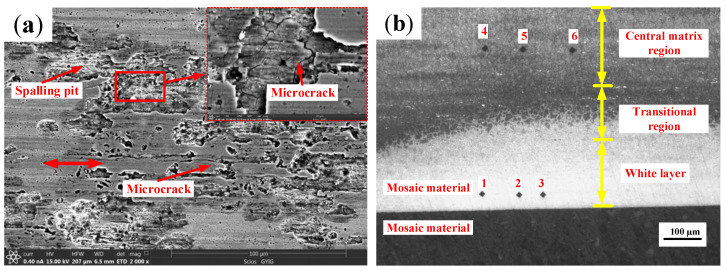
The damage surface of the inner ring on the skidding burn bearing. (**a**) Microscopic topography; (**b**) Metallographic microstructure.

**Figure 16:**
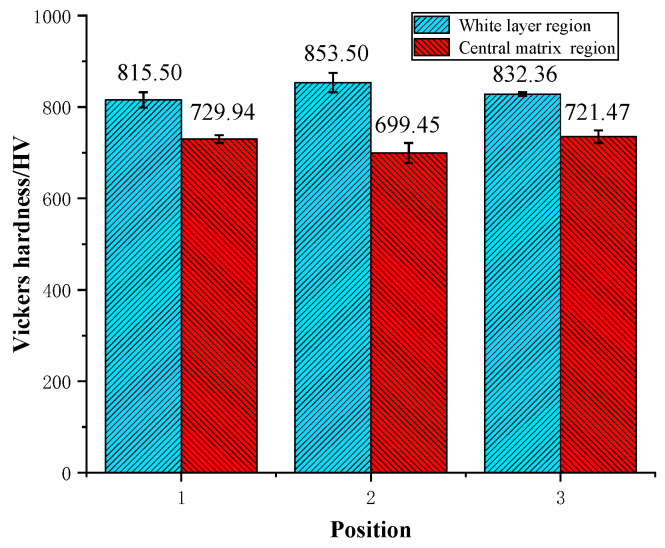
Comparison of Vickers hardness between white layer region and central matrix region on the metallographic structure of inner ring of the skidding burn bearing.

A large area of fish-scale spalling pits with different sizes and distributions were found on the damaged surface of the inner ring of skidding burn bearing, and a large number of reticular cracks appeared at the bottom, as shown in Figure 15a. A thick white layer structure was found in the damage area of inner ring, and its maximum thickness is about 180 µm, as shown in Figure 15b. A black reticular transition region appears between the white layer and the central matrix regions, and the grain boundaries can be clearly seen. Vickers hardness measurement was carried out in the white layer region and the central matrix region, and the results indicate that the hardness in the white layer region is higher than that in the central matrix region (Figure 16). Therefore, it can be seen that the bearing has suffered from secondary quenching burns, and the white layer structure is the secondary quenching martensite. This may be because the local flash temperature generated at the moment of skidding burn exceeds the phase transformation temperature of the inner ring structure, which makes the pearlite transform to the austenite, and then it is hardened by a relatively cold matrix to form the quenching martensite. It can be predicted that when the cylindrical roller bearing continues to run at a higher speed after skidding damage, the secondary quenching burn will easily occur, which has great potential safety hazards. Therefore, it is particularly important for large rotating machines to monitor the friction torque, vibration, and temperature of cylindrical roller bearings in real time during actual operation.

## 4. Conclusions

1. The cylindrical roller bearing has an instantaneous increase in the friction torque, vibration acceleration, and temperature when the skidding damage happens, and the critical speed of skidding damage is relatively low under the conditions of inadequate lubrication or a light load. The longer the running time of the bearing under the critical speed of skidding damage is the more serious the wear is. Moreover, with an increase of speed, a decrease of radial load and an increase of viscosity of lubrication oil, the slip rate of cage shows an increasing trend. A large number of pits and cracks appear on the damaged surface of the inner ring and roller.
2. The friction torque of the cylindrical roller bearing gradually increases at first and then it starts to decrease and gradually tends to stabilize with an increase in the speed, and it increases rapidly in the initial stage and then the increase trend slows down with an increase in the radial load. The vibration acceleration increases steadily along with the increase of the speed, and it should be noted that the vibration acceleration increases sharply when the speed higher than a specific value. The temperature of cylindrical roller bearing basically shows an increasing trend with the increase of the speed and the decrease of the oil intake. The greater the acceleration is, the faster the instantaneous increase of friction torque and vibration acceleration are and the greater the maximum friction torque is. The two parameters have little difference respectively under different accelerations after the speed is stable.
3. The major wear mechanisms of skidding damage of the cylindrical roller bearing includes oxidation wear, abrasive wear, and delamination wear. The white layer is found locally on the bearing surface under the action of friction heat and shear force. Subsequently, vertical and horizontal cracks are formed in the white layer, and the spalling occurs when the crack is closed with the surface.
4. Testing the running state of large rotating machines, especially vibration, bearing temperature, and friction torque, is the most effective way to avoid skidding damage or even burn for the cylindrical roller bearing.

## Figures and Tables

**Table 1:** Basic structural parameters of the test bearing.

Parameter	Value
Bore diameter of outer ring	Φ167 mm
Outer diameter of outer ring	Φ180 mm
Inner ring raceway diameter	Φ143 mm
Bore diameter of inner ring	Φ130 mm
Bore diameter of cage	Φ152 mm
Outside diameter of cage	Φ166 mm
Number of rollers	30
Roller Diameter	Φ12 mm
Roller length	12 mm

**Table 2:** Chemical element composition of Cr4Mo4V bearing steel (Mass fraction/wt.%).

Element	Fe	Mo	Cr	V	C	P	Si	Mn
Mass fraction	88.573–90.6	4.0–4.5	3.75–4.25	0.9–1.1	0.75–0.85	≤0.027	≤0.35	≤0.35

**Table 3:** Chemical element composition of silicon bronze cage (Mass fraction/wt.%).

Element	Cu	Ag	Si	Zn	Fe	Sn	Ni	Mn	P	Pb
Mass fraction	84.19–88.5	4.3–5.1	3.0–4.0	2.5–3.5	1.2–1.8	≤0.25	≤0.2	0.5–0.9	≤0.03	≤0.03

**Table 4:** The conditions for different tests.

Test Parameters	Realization of the Skidding Damage	Realization of the Skidding Burn	Slipping Dynamic Behavior	Skidding Damage Dynamic Behavior
Radial load/KN	0.2	1	0.2–4.2	0.2/1
Oil intake/(L/min)	2.166	4.332	0.5415–3.249	0.5415/2.166
Speed acceleration/(rpm/h)	3.6 × 10^5^	3.6 × 10^5^	3.6 × 10^6^/1.8 × 10^6^	3.6 × 10^5^
Load acceleration/(KN/h)	6.0 × 10^3^	6.0 × 10^3^	6.0 × 10^3^	6.0 × 10^3^
Speed/rpm	0–13,980	0–2 × 10^4^	0–1.2 × 10^4^	0–14,961
Temperature	Room temperature	Room temperature	Room temperature	Room temperature
Relative humidity/%	60 ± 5	60 ± 5	60 ± 5	60 ± 5
Atmosphere	Laboratory air	Laboratory air	Laboratory air	Laboratory air

**Table 5:** The critical speed of skidding damage for the cylindrical roller bearing.

NO	Radial Load/KN	Oil Intake/L·min^−1^	the Critical Speed of the Skidding Damage/rpm
1	0.2	2.166	13,980
2	0.2	0.5415	11,050
3	1	0.5415	14,961
4	1	2.166	14,940

**Table 6:** Calculation results of the slip rate of cage.

Speed/rpm	Radial Load/N	Viscosity of Lubricating Oil/mPa·s	the Slip Rate of Cage/%
13,985	1000	19.23	16.5
12,069	1000	19.23	15.5
13,989	300	17.48	15.8
13,989	200	17.48	50.3
14,940	1000	7.25	12
13,985	1000	19.23	16.5
